# A novel method to detect rare variants using both family and unrelated case-control data

**DOI:** 10.1186/1753-6561-5-S9-S80

**Published:** 2011-11-29

**Authors:** Tao Feng, Robert C Elston, Xiaofeng Zhu

**Affiliations:** 1 Department of Epidemiology and Biostatistics, School of Medicine, Case Western Reserve University, Wolstein Research Building, 2103 Cornell Road, Cleveland, OH 44106, USA

## Abstract

To detect rare variants associated with a phenotype, we develop a novel statistical method that can use both family and unrelated case-control data. Unlike the currently existing methods, we first use family data to calculate weights to be given to rare variants, differentiating between concordantly affected and discordant sib pairs. These weights are then used in an association test applied to the unrelated case-control data. We applied the proposed method to the simulated sequencing data in Genetic Analysis Workshop 17 and identified two genes associated with the disease.

## Background

Genome-wide association studies, which are based on the common disease/common variants assumption, have successfully identified susceptibility loci for complex traits. However, the variants discovered through these studies explain only a modest portion of the trait variability [1]. With the new technological advances, it has been suggested that it is time to shift the search from common variants of modest effect to rarer variants of large effect by effectively searching the full genome [2]. Rare variants may hold promise to predict individual risk for personalized medicine because of their large effect, although it has been argued that common variants illuminate the biologic pathways that underlie diseases [3].

Bodmer and Tomlinson [4] suggested that a set of low-frequency variants from different genes can account for a significant proportion of the variability of relatively common diseases. To achieve reasonable statistical power, it is critical to define the rare variants and test them collectively. The existing statistical methods in the literature mainly collapse rare variants [5]. Madsen and Browning [6] proposed using the inverse of the variance of the minor allele frequency (MAF) in control subjects as a weight and then collapsing the weighted rare variants.

Briefly, for the *i*th individual Madsen and Browning [6] define a genetic score:(1)

where *L* is the number of variants, *g_ij_* is the genotypic score, and *w_j_* is the weight for the *j*th single-nucleotide polymorphism (SNP). The weight *w_j_* is defined as the inverse of the *j*th SNP’s standard deviation estimated in control subjects when the corresponding MAF is less than *α* (such as 0.02) and 0 otherwise. Then the Wilcoxon rank sum test is applied to do the association test. Madsen and Browning rank the genetic scores, calculate the sum of the ranks for case subjects as:(2)

and calculate the *p*-value using a permutation strategy. That is, they permute disease status among individuals 1,000 times to compute 1,000 statistics *X*, denoted , , …, . Then they use the sample mean  and the standard deviation  of , , …,  to calculate the test statistic:(3)

which follows approximately a standard normal distribution under the null hypothesis. Madsen and Browning [6] demonstrated that this weighted-sum method is more powerful than the collapsing method [5].

Recently, we have demonstrated that family data are useful for searching for rare variants [7,8], because the rare variants can be substantially enriched among segregating family members. Here, we present a statistical method to test rare variants by using both family and unrelated case-control sequencing data.

## Methods

### Defining a weight for each SNP using the family data set

We start by assuming that we have *L* variants belonging to a group (gene, pathway, specific genomic region, etc.). We have shown that family data, such as affected sib pairs, have enriched information for detecting rare disease-associated variants because the same disease variants are more likely to segregate within a family [7]. Thus each family can be considered homogeneous; that is, affected family members share the same or allelic disease variants, the latter being an idea used in traditional linkage analysis. Our method uses either affected sib pairs or discordant sib pairs to determine weights for such rare variants.

Assume that a SNP has two alleles *A* and *a*, where *A* always refers to the minor allele. For the *i*th sib pair and the *j*th SNP, we define a genotype score . Let *α* be a predefined threshold. We always let  if a SNP has a MAF greater than *α*. For those SNPs with MAF ≤ *α* , we define  as follows. For affected sib pairs,  if both sibs carry *A*,  if neither sib carries *A*, and  if one sib carries *A* and the other does not, where *K* is the number of other SNPs in the region with MAF <*α* carried by the other sib. For discordant sib pairs,  if the affected sib carries allele *A* and the unaffected sib does not,  if the affected sib does not carry *A*, and  if both sibs carry *A*, where *K* is the number of alleles with MAF <*α* present at the other SNPs carried by the affected sib.

Let:(4)

where *N*_sib_ is the number of sib pairs. For the *j*th SNP, define:(5)

where *p_j_* is the MAF of the SNP, estimated from the case and control subjects combined. We rank the *b_j_* in descending order and give the *j*th SNP weight *w_j_* = *b_j_* if *b_j_* falls in the top quartile and weight *w_j_* = 0 if not. The proposed weight incorporates information about the variants shared (not shared) by affected (discordant) sib pairs.

### Performing association tests

After defining the weights for individual SNPs, using either affected or discordant sib pairs, we use the weights to test for association between the phenotype and each set of variants in a group. We assume that we have *N_D_* unrelated case subjects and *N_C_* unrelated control subjects. Let *k* be the *k*th unrelated individual. For each SNP *j* (*j* = 1, …, *L*), we let *w_j_* be the weight as found earlier using the sib pairs. We use the *w_j_* to calculate the *k*th individual’s genetic score, similar to the idea of Madsen and Browning [6]; that is,(6)

where *g_kj_* is the number of minor alleles in SNP *j* for individual *k*. We then define:(7)

where the summation is over the unrelated case subjects. Similarly, we define:(8)

calculated for the unrelated control subjects. Our null hypothesis is that no marker is associated with the phenotype; that is, we have , and so we define our test statistic for association as:(9)

where  and  are estimates of the sample variances  and  in case and control subjects, respectively.

### Application to the Genetic Analysis Workshop 17 sequence data set

The Genetic Analysis Workshop 17 (GAW17) simulated sequence data set includes both family and unrelated individuals data, which is ideal for the proposed method. Each replicate is composed of 697 individuals in the family data set and 697 individuals in the unrelated individuals data set. There are 200 replicates in total, all with the same genotype data but with phenotype data independently simulated across the replicates.

We first performed an analysis using only one replicate, which was selected to be the 82nd replicate. To calculate the weights, we clustered the sib pairs into affected and discordant sib pairs. Based on the 82nd phenotype replicate, we identified 38 affected and 22 discordant sib pairs. We then set *α* = 0.01 as the MAF cutoff to define the rare variants and calculated weights based on the identified 38 affected sib pairs using the Eq. (4-5).

The 82nd phenotype replicate includes 209 unrelated case subjects and 488 unrelated control subjects. This data set was used to test for association in each of 3,205 genes.

We compared the power of the proposed method with Madsen and Browning’s method using the same unrelated case-control data. The same MAF threshold of *α* = 0.01 was used to define rare variants for Madsen and Browning’s method.

We expect there to be little or no power for detecting rare variants using a single replicate because of the small sample size. We therefore included additional replicates until we reached a sample size of 400 affected sib pairs and 2,400 case subjects and 2,400 control subjects. We used 400 affected sib pairs, which was suggested by our previous study [7]. Although the genotypes are the same for the different replicates, the genotype-phenotype association is simulated independently for the different replicates. Thus the way we increased the sample size will have little impact on our power comparison.

## Results

We applied the proposed method and Madsen and Browning’s method to 3,205 genes using the 82nd replicate. As expected, we found virtually no power for either method.

**Figure 1:**
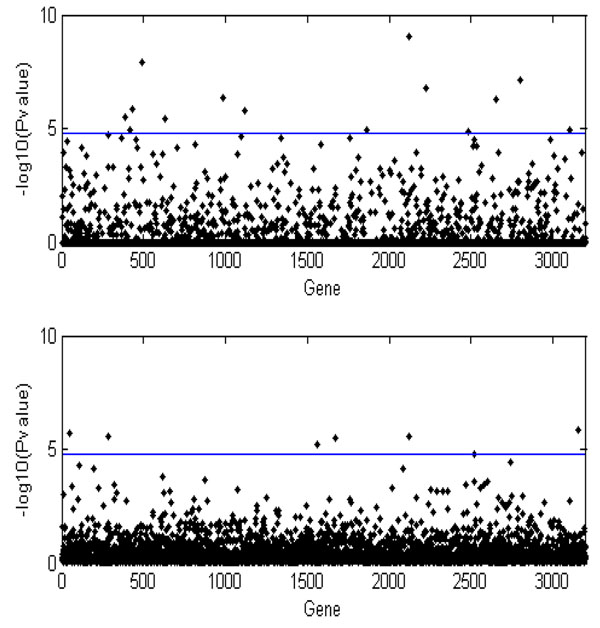
**Manhattan plot of –log_10_(*p*-value) for each single gene after pooling data to reach a larger sample size** The upper panel is the result of our proposed method, and the lower panel is the result of Madsen and Browning’s method.

We next increased the sample size to 400 affected sib pairs to calculate the weights and an additional 2,000 case subjects and 2,000 control subjects for the association test using our proposed method. For comparison, we used 2,400 case subjects and 2,400 control subjects for Madsen and Browning’s method. Thus the total sample size is the same for both methods. Figure 1 presents the result for testing 3,205 genes. The horizontal line indicates the 5% significance level after adjusting for 3,205 tests. After correcting for multiple comparisons, we observed 14 and 7 genes reaching significance for the proposed and Madsen and Browning’s methods, respectively; 2 of 14 and 1 of 7 significant genes are real causal signals.

We next examined the 15 causal genes generated in the simulations (Table 1). Both methods detected *PICK3C2B*, but our proposed method resulted in a much smaller *p*-value (8.67 × 10^−10^ vs. 1.48 × 10^−5^). Furthermore, the proposed method identified *HSP90AA1* (*p* = 8.63 × 10^−6^), which was missed by Madsen and Browning’s method.

**Table 1:** Causal gene with tested *p*-value

Gene	*p*-value (Madsen and Browning method)	*p*-value (our method)
*AKT3*	0.173	1.0
*BCL2L11*	0.0555	1.0
*ELAVL4*	0.90	0.0283
*HSP90AA1*	0.737	8.63 × 10^−6^
*NRAS*	0.459	1.0
*PIK3C2B*	1.48 × 10^−5^	8.67 × 10^−10^
*PIK3C3*	0.796	1.0
*PIK3R3*	0.732	0.236
*PRKCA*	0.564	1.0
*PRKCB1*	0.684	0.236
*PTK2*	0.868	1.0
*PTK2B*	0.433	1.0
*RRAS*	0.480	1.0
*SHC1*	0.925	1.0
*SOS2*	0.465	1.0

The causal genes of latent liability disease and their *p*-values found using Madsen and Browning’s method and our proposed method.

## Discussion and conclusions

In this paper, we propose a novel method to analyze the GAW17 data set. Unlike the existing methods, the proposed method calculates the weights using either affected or discordant sib pairs. The proposed method requires that both the case and control groups and the family members are genotyped for the same set of SNPs or are resequenced in the same region. Compared with Madsen and Browning’s method, the proposed method detects the true causal gene *HSP90AA1*, which was missed by Madsen and Browning’s method. Our method demonstrates that incorporating family data can potentially improve statistical power to detect rare variants in an association analysis, which is consistent with the result of Zhu et al. [7].

## Competing interests

The authors declare that there are no competing interests.

## Authors’ contributions

XZ designed the study. TF performed the data analysis. TF and XZ drafted the manuscript. RCE revised the manuscript. All authors read and approved the final manuscript.
